# Distinct delay discounting patterns in anorexia nervosa: Comparing monetary and exercise rewards across clinical subgroups

**DOI:** 10.1111/pcn.13802

**Published:** 2025-02-10

**Authors:** Paolo Meneguzzo, Luisa Ladduca, Pietro Balducci, Valentina Meregalli, Enrica Bucci, Anna Pillan, Chiara Cazzola, Alice Garolla, Elisa Bonello, Francesca Buscaglia, Patrizia Todisco

**Affiliations:** ^1^ Department of Neuroscience University of Padova Padova Italy; ^2^ Padova Neuroscience Center University of Padova Padova Italy; ^3^ Eating Disorder Unit Casa di Cura “Villa Margherita” – Neomesia Arcugnano Italy

**Keywords:** anorexia nervosa, compulsivity, delay discounting, impulsivity, physical activity

## Abstract

**Aims:**

People with anorexia nervosa (AN) often engage in compulsive exercise to control weight and manage emotions, altering the reward associated with physical activity. Delay‐discounting evaluates preference for immediate over delayed rewards, potentially indicating struggles with prioritizing long‐term gains. However, the impact of AN on these factors remains unexplored. This study aims to assess the attitudes of individuals with AN using a modified exercise delay discount task, comparing them with the general population.

**Methods:**

A sample of 76 individuals (70 women, 92.1%) with a diagnosis of AN was compared with a sample of 124 people (115 women, 92.7%) without a lifetime diagnosis of any eating disorder. Two different delay discount tasks were used to evaluate different constructs: a standardized monetary delay discount task and a modified version focused on physical activity.

**Results:**

The standardized monetary delay discount task confirmed the existence of varied monetary rewards in different subtypes of AN. The exercise‐related task demonstrated a specific emphasis on exercise within AN, showing a tendency to delay physical activity by looking for a greater amount of exercise. On the contrary, the general population opted for immediate exercise proposals.

**Conclusions:**

Our findings suggest that the tendency to delay physical activity could be related to the compulsive nature of exercise in patients with AN, with a possible change in cognitive evaluation based on the magnitude of exercise. Finally, in addition to psychological problems related to eating disorders, additional cognitive factors likely contribute to exercise compulsiveness, necessitating further research exploration.

Anorexia nervosa (AN) is a serious eating disorder (ED) marked by an intense fear of weight gain, resulting in severe food restrictions, distorted body image, and, frequently, excessive physical activity.^1^, ^2^ Approximately 30–80% of people with AN engage in compulsive exercise,^3^, ^4^ which is recognized as a significant factor in the etiology, development, and maintenance of EDs, particularly AN.^5^ Compulsive exercise often involves rigid schedules and prioritization of activity over other tasks, reflecting a maladaptive, habitual behavior. Several studies have highlighted that the presence of excessive levels of physical activity is associated with longer hospitalizations,^6^ an increased risk of suicidal behaviors,^7^ and a higher likelihood of relapse and long‐term EDs.^8^, ^9^, ^10^, ^11^ However, this aspect remains poorly understood.

Anorexia nervosa is characterized by two main subtypes: restrictive (ANr), which involves significant food restrictions, and binge‐purge (ANbp), where individuals engage in overeating followed by compensatory behaviors such as self‐induced vomiting or purging.^9^, ^10^ Previous research has highlighted that impulsivity and compulsivity play distinct roles in these subtypes. Impulsivity refers to a tendency to prioritize immediate rewards, even at the expense of long‐term consequences.^10^, ^11^ In ANbp, higher levels of impulsivity are often associated with a preference for immediate rewards, as seen in binge‐eating behavior, where the immediate gratification of eating outweighs the potential long‐term negative consequences.^11^ Compulsivity, on the other hand, involves repetitive, maladaptive behaviors driven by the need to reduce anxiety or discomfort, despite no immediate reward.^12^, ^13^ In ANr, compulsivity is more prominent, and individuals with this subtype tend to favor delayed, larger rewards over immediate ones, as reflected in the compulsive nature of physical activity. These individuals exhibit rigid, habitual behaviors, such as excessive exercise, often as a way to delay or prevent weight gain, reflecting greater self‐control and a longer‐term focus.^13^, ^14^

This dissociation between impulsivity and compulsivity in AN subtypes might be conceptualized through the lens of delay discounting.^11^, ^15^, ^16^ In the case of impulsivity (more prominent in ANbp), individuals discount future rewards more steeply and prefer immediate, smaller rewards (e.g. binge eating for immediate relief). In contrast, individuals with compulsive behaviors (more characteristic of ANr) demonstrate a tendency to value delayed rewards more highly, often engaging in maladaptive behaviors like excessive exercise, which provide long‐term, albeit unhealthy, benefits in terms of body image and weight control. Thus, the relationship between impulsivity and compulsivity in AN might not be straightforward; rather, they are related but distinct dimensions, with each influencing decision‐making and behavior in different ways across the AN subtypes.^14^, ^17^, ^18^, ^19^

Delay discounting is a theoretical framework that describes how sensitivity to immediate rewards decreases as the delay in their delivery increases.^20^ It assesses choice patterns where the value of a reward diminishes (or is discounted) as the waiting time for its delivery grows. This framework can also be applied to understanding responses to distress‐provoking obsessions through compulsive behavior. Essentially, a compulsion can be seen as the selection of short‐term rewards (e.g. immediate reduction of anxiety) at the expense of long‐term rewards (e.g. living a more meaningful life). The concept is often studied in decision‐making scenarios where people are presented with the choice between receiving a smaller amount of something (e.g. money, food) immediately or a larger amount after a delay.^21^ In EDs, different subtypes exhibit distinct patterns of monetary delay discounting. Higher levels of impulsivity, associated with disorders of the bulimic spectrum such as bulimia nervosa, binge ED, and ANbp, are linked to a preference for immediate rewards. On the contrary, ANr is associated with caution and a tendency to choose delayed rewards, reflecting a markedly elevated level of self‐control.^11^ In different clinical scenarios, such as obsessive‐compulsive disorder, delay discounting has been used with novel tasks such as hand washing, showing that these symptom‐specific delay discounting tasks can help elucidate the relationship between impulsivity and psychological conditions.^22^ Therefore, in this study, we developed a novel exercise delay discounting task to assess how this aspect might vary among different subtypes of AN.

This study aimed to investigate the phenomenon of delayed discounting with respect to exercise in people with AN, shedding light on the cognitive evaluation of this facet of behavior. The primary hypothesis posited that individuals with AN would exhibit a differential discount of rewards associated with exercise compared to the general population, attributable to the unique significance of exercise within the context of AN. Specifically, we hypothesized that people with ANr would demonstrate a preference for compulsive, delayed, greater rewards over smaller, immediate rewards in the realm of exercise‐related decisions, reflecting increased compulsivity in this clinical population, compared to individuals with ANbp, who may exhibit more impulsive choices.

## Methods

### Sample

The sample consisted of 76 white cisgender patients (70 female, 92.1%) admitted to the Emergency Department of the Villa Margherita Clinic in Arcugnano (Italy), between 16 and 40 years old. Among them, 41 received a diagnosis of ANr and 35 received a diagnosis of ANbp. Diagnoses were formulated by a trained psychiatrist using structured clinical interview for DSM5.^23^

These patients were compared with a control group consisting of 124 white cisgender subjects (115 female, 92.7%) between 16 and 44 years old and enrolled through a public call for volunteers in psychological experiments through social media pages. The excluded criteria for community participants (CP) included a history of ED and the presence of any other psychiatric conditions evaluated by a trained researcher through an online interview.

Participants in the CP group were not subject to any restrictions regarding exercise or dietary intake. They were free to engage in regular physical activity and consume food as they chose throughout the study period. For participants in the AN group, strict dietary restrictions were not permitted. All participants in this group were required to adhere to a structured recovery plan, which included regular nutrition intake to prevent starvation. Exercise was controlled but allowed; participants were permitted to engage in light physical activity, such as walking around the structure or performing low‐intensity exercises, in line with their treatment plan. These restrictions were in place to prevent excessive exercise, while still allowing for the evaluation of delayed exercise rewards within a controlled framework.

All participants gave their written informed consent during their recruitment. If a participant was under 18 years old, consent was collected from their parents. The study protocol was in accordance with the ethical standards of the Declaration of Helsinki and its subsequent amendments and was approved by the Vicenza Ethics Committee (47/21).

### Self‐report measures

Demographic data was collected for all participants such as age, height, and weight to evaluate body mass index (BMI). Different self‐report questionnaires were also collected before performing the delay discounting task.

The Eating Disorder Examination Questionnaire is a well‐established self‐report instrument that measures ED behaviors and attitudes.^24^ It is composed of 28 items and is scored on a 7‐point Likert scale. It is categorized into four subscales (restraint, eating concern, shape concern, and weight concern) and an overall global score, with a higher score indicating more problematic eating difficulties. In the current study the Cronbach's α were comprised between 0.91 and 0.94. The short version of the UPPS‐P impulsive behavior scale (S‐UPPS‐P) is a widely used measure of impulsivity.^25^ It consists of 20 items scored on a four‐point Likert scale and it is composed of five subscales. Negative urgency, positive urgency, lack of premeditation, lack of persistence, and Sensation Seeking. Higher scores on certain facets may indicate a greater propensity toward impulsive behavior in those specific domains, while lower scores suggest less impulsivity in those areas. In the current study, the Cronbach's α was between 0.83 and 0.90.

The compulsive exercise test (CET) is a self‐report questionnaire designed to assess the tendencies toward compulsive exercise, with higher scores indicating higher levels of compulsive exercise behavior.^26^ It is composed of 24 items scored on a 6‐point Likert‐type scale and generates a global score and five subscales: Avoidance and rule‐driven behavior, weight control exercise, mood improvement, lack of exercise enjoyment, and exercise rigidity. A cut‐off score for identifying compulsive exercisers has been proposed at 15 points.^27^ In the current study, the Cronbach's α was between 0.78 and 0.88.

### Delay discounting tasks

In the present study, two different delay discounting tasks were used: the first involving monetary choices (27 items) and the second replacing money with physical activity (27 items). The initial task replicated a validated monetary choice task^11^, ^28^ already applied to assess this construct in the context of EDs. The second task, tailored for this study, maintained the 27 items from the original task by Kirby *et al*. but replaced money with minutes of physical activity. Physical activity served as a reward, as suggested in the literature,^29^ measuring the levels of delay discounting associated with physical activity (for example, ‘Would you prefer to exercise 31 minutes of exercise now or 85 minutes in 7 days?’). These tasks measure individual intertemporal discount rates (*k*) by presenting a series of choices between a smaller, immediate monetary reward and a larger, delayed monetary reward. Each question is crafted to align with a specific *k*‐value, reflecting the degree of discount applied to the future reward to make it equivalent to the immediate one. Scoring is based on where the respondent's choices fall among reference discounting curves, with placement on steeper curves indicating higher impulsivity in decision‐making. Single *k* parameter estimates can be derived to represent the overall rate of discounting, as well as for small, medium, and large rewards (Kirby *et al*.,^28^). The overall values of *k* range from 0 (as always choosing the delayed reward, indicating that there is no discount) to 0.25 (as always choosing the immediate reward). Previous studies have demonstrated a magnitude effect on discount rates, where *k* values decrease as the reward amount increases. Thus, *k* values were separately estimated using three reward magnitude categories^30^: small delayed rewards (25–35 euros or minutes), medium delayed rewards (50–60 euros or minutes).

### Statistical analyses

Nonparametric analyzes were applied due to the distribution of the majority of the variables included, with Kruskal‐Wallis one‐way analysis, followed by *post hoc* analysis with Bonferroni correction to assess differences between groups. The study delved into potential correlations between the subscales of the UPPS‐P and CET questionnaires and the *k*‐values, using Spearman's rank correlation coefficient (ρ) separately for patients and CP. Potential correlations between *k*‐values across different tasks were also examined to investigate any shared patterns.

To assess the consistency of delay discounting behavior across two tasks in participants, we calculated the intraclass correlation coefficient (ICC) using the three *k*‐values representing discounting rates for large, medium, and small rewards. The ICC was computed for absolute agreement based on a two‐way mixed‐effects model, reflecting the degree to which individual participants' discounting rates were consistent across the two tasks. This approach allowed us to account for both individual differences in delay discounting and task‐specific variability. Separate analyses were performed to compare consistency within the AN group, the CP group, and the entire sample to examine whether group differences influenced the observed agreement. The comparison between the subgroups' ICCs was evaluated using Fisher's *Z*‐transformation, with ±1.96 as the critical value of *Z*_diff_ for significance.^31^

A two‐way analysis of variance (anova) was performed, examining the interaction effects of compulsive exercise and group. *Post hoc* pairwise comparisons with Bonferroni correction were conducted to explore group differences when significant interactions were detected.

Statistical analysis was performed using IBM SPSS Statistics version 25 (IBM Corp., 2017) and the significance was set to α <0.05 due to the exploratory nature of the study.

## Results

### Comparisons between groups

There were no significant differences between the clinical and control groups for age. Clinical groups reported significantly lower BMI and significantly higher scores on all self‐report questionnaires, with differences in impulsivity between ANr and ANbp, according to the literature. The mean scores, standard deviations, and group comparisons for each of the measure subscales are presented in Table 1.

**Table 1:** Demographic and psychological characteristics of the participants

Variables	CP	ANr	ANbp	*Z*	*P*	*Post hoc* (*P*)
Age, years	21.19 (3.28)	20.71 (5.36)	21.60 (4.54)	5.304	0.071	
BMI, kg/m^2^	21.20 (2.89)	15.25 (1.58)	15.77 (0.96)	128.894	<0.001	CP > ANr (<0.001) CP > ANbp (<0.001)
EDEQ						
Restraint	0.87 (1.06)	3.43 (1.42)	4.16 (1.92)	94.166	<0.001	CP < ANr (<0.001) CP < ANbp (<0.001)
Eating concern	0.42 (0.75)	2.90 (1.08)	4.19 (1.22)	131.892	<0.001	CP < ANr (<0.001) CP < ANbp (<0.001)
Shape concern	1.67 (1.35)	4.46 (1.19)	5.27 (1.04)	113.467	<0.001	CP < ANr (<0.001) CP < ANbp (<0.001)
Weight concern	1.41 (1.35)	3.69 (1.20)	4.67 (1.36)	98.326	<0.001	CP < ANr (<0.001) CP < ANbp (<0.001)
Global score	1.09 (0.99)	3.64 (1.01)	4.57 (1.22)	122.164	<0.001	CP < ANr (<0.001) CP < ANbp (<0.001)
S‐UPPS‐P						
Positive urgency	8.95 (3.40)	9.83 (3.85)	12.03 (3.08)	21.217	<0.001	CP < ANbp (<0.001) ANr < ANbp (0.020)
Negative urgency	8.57 (3.29)	8.98 (3.13)	10.23 (3.76)	6.150	0.046	CP < ANbp (0.042)
Lack of premeditation	6.29 (2.26)	7.37 (1.76)	8.94 (2.84)	29.590	<0.001	CP < ANr (0.022) CP < ANbp (<0.001)
Lack of perseverance	6.00 (2.15)	7.00 (2.87)	9.51 (1.93)	50.477	<0.001	CP < ANbp (<0.001) ANr < ANbp (0.020)
Sensation seeking	8.41 (3.60)	9.80 (4.14)	11.37 (3.40)	17.945	<0.001	CP < ANbp (<0.001)
CET						
Avoidance	1.47 (0.81)	2.55 (2.15)	2.41 (1.64)	12.014	0.002	CP < ANr (0.017) CP < ANbp (0.023)
Weight control	2.45 (0.97)	3.16 (1.38)	3.60 (1.53)	24.979	<0.001	CP < ANr (0.006) CP < ANbp (<0.001)
Mood improvement	1.48 (1.24)	3.37 (1.31)	3.22 (1.39)	63.040	<0.001	CP < ANr (<0.001) CP < ANbp (<0.001)
Lack of exercise enjoyment	2.16 (0.82)	2.12 (0.83)	1.90 (1.03)	6.025	0.049	CP < ANbp (0.045)
Exercise rigidity	1.58 (1.07)	2.97 (1.01)	2.47 (1.43)	40.029	<0.001	CP < ANr (<0.001) CP < ANbp (0.002)
Global score	9.63 (3.13)	14.36 (4.58)	13.60 (5.12)	42.070	<0.001	CP < ANr (<0.001) CP < ANbp (<0.001)

AN, anorexia nervosa; BMI, body mass index; bp, binge‐purge; CET, compulsive exercise test; CP, community participant; EDEQ, Eating Disorder Examination Questionnaire; r, restrictive; S‐UPPS‐P, short version of the impulsive behavior scale.

**Fig. 1:**
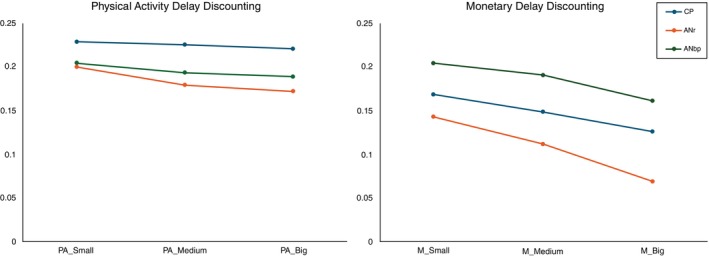
Mean discount rate (y‐axis) as a function of delayed reward magnitude (x‐axis). AN, anorexia nervosa; bp, binge‐purge; CP, community participant; PA, physical activity; r, restrictive. Blue line: Community Participant (CP), red line: Anorexia Nervosa restrictive (ANr), green line: anorexia nervosa binge‐purge (ANbp).

**Fig. 2:**
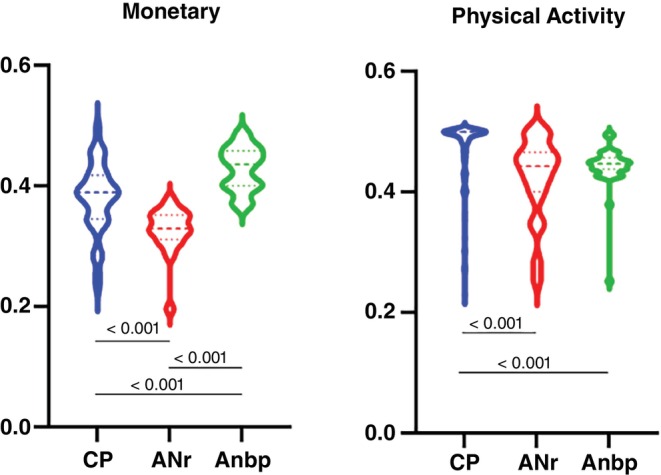
Violin plot for overall *k* values. Blue line: Community Participant (CP), red line: Anorexia Nervosa restrictive (ANr), green line: anorexia nervosa binge‐purge (ANbp).

Concerning the delay discounting task, distinct outcomes emerged between the money and physical activity tasks. In the money delay discounting, differences were observed across all groups. Individuals with ANr showed lower levels of delay discounting, as evidenced by the smallest *k*‐values, whereas those with ANbp exhibited higher delay discounting compared to CP with more steep choices. On the delays in contrast, in the physical activity, disparate findings emerged. ANbp and ANr reported similar decisions with *k*‐values lower than CP, while CP consistently reported an immediate choice in all scenarios. Refer to Table 2 for detailed results and Figure 1 for a graphical representation of the different *k*‐values. In Figure 2 we reported the means of the *k*‐values to represent the distributions of the average choices.

**Table 2:** Delay discounting tasks results

Tasks	CP	ANr	ANbp	*Z*	*P*	*Post hoc* (*P*)
Money						
Big	0.13 (0.05)	0.07 (0.03)	0.16 (0.05)	61.866	<0.001	ANr < CP (<0.001) CP < ANbp (0.005) ANr < ANbp (<0.001)
Medium	0.15 (0.05)	0.11 (0.03)	0.19 (0.03)	54.941	<0.001	ANr < CP (<0.001) CP < ANbp (<0.001) ANr < ANbp (<0.001)
Small	0.17 (0.04)	0.14 (0.03)	0.20 (0.03)	50.284	<0.001	ANr < CP (<0.001) CP < ANbp (<0.001) ANr < ANbp (<0.001)
Average	0.15 (0.04)	0.11 (0.02)	0.19 (0.03)	46.055	<0.001	ANr < CP (<0.001) CP < ANbp (<0.001) ANr < ANbp (<0.001)
Physical activity						
Big	0.22 (0.05)	0.17 (0.07)	0.19 (0.04)	42.843	<0.001	CP > ANr (<0.001) CP > ANbp (<0.001)
Medium	0.22 (0.05)	0.18 (0.06)	0.19 (0.03)	52.115	<0.001	CP > ANr (<0.001) CP > ANbp (<0.001)
Small	0.23 (0.04)	0.20 (0.05)	0.20 (0.04)	37.642	<0.001	CP > ANr (<0.001) CP > ANbp (<0.001)
Average	0.22 (0.05)	0.18 (0.05)	0.20 (0.03)	48.510	<0.001	CP > ANr (<0.001) CP > ANbp (<0.001)

AN, anorexia nervosa; bp, binge‐purge; CP, community participant; r, restrictive.

### Agreement between tasks

The ICC analysis revealed excellent agreement across the two delay discounting tasks for the entire sample (ICC = 0.76, 95% CI: 0.71–0.81, *P* <0.001), suggesting that participants' discounting rates were relatively consistent between monetary and physical activity contexts. Subgroup analyses showed comparable agreement in the CP group (ICC = 0.79, 95% CI: 0.71–0.83) and AN group (ICC = 0.71, 95% CI: 0.59–0.80), with no significant *Z*_diff_ (0.42). Confidence intervals in both groups indicated moderate reliability but suggested some variability in individual responses between tasks. Looking at the AN subgroups, we found moderate agreement in restrictive AN (ICC = 0.63, 95% CI: 0.13–0.63), comparable to controls (*Z*_diff_ = 0.75), and poor agreement in binge‐purge AN (ICC = 0.35, 95% CI: −0.04–0.60, *Z*_diff_ = 1.42).

### Correlation analyses

Looking at the relationship between the two delay discounting tasks, we found no association between them in all the samples. There were strong connections between different magnitudes of *k* values within the tasks; see Table 3 for details.

**Table 3:** Spearman correlation analyses between delay discounting tasks

Variables	PA big	PA medium	PA small	PA average	M big	M medium	M small	M average
CP								
PA big	—	0.785**	0.627**	0.907**	0.085	0.066	0.066	0.093
PA medium	0.785**	—	0.734**	0.811**	0.105	0.018	0.031	0.078
PA small	0.627**	0.734**	—	0.903**	0.089	0.058	0.051	0.084
PA average	0.907**	0.811**	0.903**	—	0.088	0.022	0.021	0.072
M big	0.085	0.105	0.089	0.088	—	0.813**	0.677**	0.903**
M medium	0.066	0.018	0.058	0.022	0.813**	—	0.842**	0.965**
M small	0.066	0.031	0.051	0.021	0.677**	0.842**	—	0.874**
M average	0.093	0.078	0.084	0.072	0.903**	0.965**	0.874**	—
ANr								
PA big	—	0.790**	0.504**	0.863**	−0.292	0.086	−0.048	−0.118
PA medium	0.790**	—	0.637**	0.933**	−0.133	0.117	−0.158	−0.020
PA small	0.504**	0.637**	—	0.746**	−0.331	0.071	−0.002	−0.073
PA average	0.863**	0.933**	0.746**	—	−0.225	0.143	−0.104	−0.039
M big	−0.292	−0.133	−0.331	−0.225	—	0.436**	0.380**	0.765**
M medium	0.086	0.117	0.071	0.143	0.436**	—	0.486**	0.843**
M small	−0.048	−0.158	−0.002	−0.104	0.380**	0.486**	—	0.552**
M average	−0.118	−0.020	−0.073	−0.039	0.765**	0.843**	0.552**	—
ANbp								
PA big	—	0.796**	0.408**	0.698**	−0.205	−0.168	−0.085	−0.135
PA medium	0.796**	—	−0.214**	0.814**	−0.020	−0.357	−0.300	−0.179
PA small	0.408**	−0.214**	—	0.162**	0.322	0.173	0.014	0.197
PA average	0.698**	0.814**	0.162**	—	−0.063	−0.262	−0.165	−0.177
M big	−0.205	−0.020	0.322	−0.063	—	0.759**	0.459**	0.872**
M medium	−0.168	−0.357	0.173	−0.262	0.759**	—	0.693**	0.928**
M small	−0.085	−0.300	0.014	−0.165	0.459**	0.693**	—	0.778**
M average	−0.135	−0.179	0.197	−0.177	0.872**	0.928**	0.778**	—

** *P* < 0.01.

AN, anorexia nervosa; bp, binge‐purge; CP, community participant; M, monetary; PA, physical activity; r, restrictive.

We also examined the associations between the two delayed discounting tasks and the psychological characteristics included in the study. In the CP group, we identified positive correlations between mood improvement, exercise rigidity, the CET global score, and the physical activity delay discounting task. Furthermore, the rigidity of the exercise also showed a positive correlation with the money delay discounting task. In the AN groups, we observed negative correlations between lack of exercise enjoyment and physical activity delay discounting for both groups. Furthermore, only the ANr group exhibited positive correlations between money delay discounting and positive urgency, negative urgency, and sensation seeker. See Table S1 for details.

### Compulsive exercise

The CET cut‐off of 15 points was used to identify individuals engaging in compulsive exercise and evaluate differences between those above and below the threshold. Among participants with ANr, 18 out of 41 individuals (43.9%) scored above the cut‐off, compared to 10 out of 35 (28.6%) in the ANbp group, and 11 out of 113 (9.7%) in the CP group. Analysis revealed a significant interaction between compulsive exercise and group for the *k*‐values of the PA delay discounting task only for large (*F* = 5.29, *P* = 0.006) *k*‐values. *Post hoc* comparisons indicated that both ANr (*P* <0.001) and ANbp (*P* = 0.006) groups significantly differed from the CP group on these *k*‐values, while no differences were found between the two AN subgroups (*P* = 0.463).

## Discussion

The present study aimed to investigate delay discounting concerning exercise in individuals with AN, shedding light on the cognitive evaluation of this facet of behavior. Our approach involved using a validated delay discounting task to complement a novel task focusing on physical activity to explore different constructs.^11^ We hypothesized that individuals with AN would exhibit differential discounting of the rewards associated with exercise compared to the general population, reflecting the unique significance that exercise holds within the context of AN.

Consistent with our hypotheses, the findings revealed distinct patterns of delay discounting between individuals with AN and the control group, particularly with choices in monetary and physical activity. Individuals with ANr displayed lower levels of delay discounting in the monetary task, indicative of a preference for delayed, higher rewards over smaller, immediate rewards. This finding is consistent with previous research suggesting increased self‐control in individuals with ANr.^11^, ^32^, ^33^ On the contrary, those with ANbp exhibited higher levels of delay discounting, indicative of a preference for immediate rewards, consistent with impulsivity often associated with bulimic‐spectrum disorders.^34^, ^35^ The use of the monetary task aimed to validate our delay discounting methodology in different contexts, as previously demonstrated with food‐ and non‐food‐related tasks.^36^, ^37^ The data obtained with the monetary task corroborated this aspect, showing that AN clinical subgroups have to be separated in studies that implicate impulsivity features.^32^, ^38^ Furthermore, the lack of significant correlation between the two delay discounting tasks across the entire sample, despite a high ICC, suggests that they may measure distinct constructs. The high ICC indicates that participants' decision‐making processes are consistent across both monetary and physical activity tasks, yet the lack of correlation reflects the domain‐specific nature of impulsivity and how it may differ when applied to different contexts, such as monetary versus exercise‐related rewards.^39^, ^40^ The lower ICC observed in the ANbp subgroup suggests greater variability in delay discounting responses across monetary and physical activity contexts, potentially reflecting the impulsivity and emotional dysregulation characteristic of this group. This finding underscores the need for further research into how these traits may differentially affect decision‐making processes in binge‐purge presentations of AN.

Interestingly, when evaluating delay discounting in the context of physical activity, a nuanced picture emerged. The ANr and ANbp groups displayed lower delay discounting compared to the control group, indicating a preference for delayed exercise rewards. This finding suggests that people with AN may value exercise differently from monetary rewards, prioritizing the long‐term benefits of physical activity (such as more calories burned) despite the immediate effort required. Similar response patterns observed in both subtypes of AN suggest that certain aspects of compulsive behavior related to physical activity may be common in different presentations of the disorder,^26^ and more studies are needed to understand the underlying mechanisms and treatment approaches for AN. In this study, we enroll people in the acute phase of the disorder, which could have influenced the results, making them more focused on greater amounts of exercise.^41^ Furthermore, the consistent preference for immediate rewards in the control group, particularly evident in the physical activity task, underscores the potential role of reward sensitivity and valuation in driving exercise behaviors. When comparing our results with the existing literature, a difference in the discounting of exercise in the general population emerged,^42^ with more impulsive choices in our sample. This could be due to the different approaches to exercise in our task, which was less specific than the other, or to the use of different ranges of time and delay. Furthermore, we should consider the possible lack of motivation to exercise, which may differ from clinical populations, or the fact that people may prioritize immediate stress relief or increased energy through shorter exercise sessions.^43^, ^44^ This aspect should be evaluated in future studies.

The correlation analyses further elucidated the relationship between delay discounting and psychological characteristics within each group, highlighting both shared and divergent patterns across the general population and individuals with AN. In the general population, the positive associations found suggest that individuals who display higher impulsivity or more rigid exercise behaviors are more likely to prioritize immediate rewards over delayed ones, indicating a stronger tendency to engage in behaviors that offer immediate reinforcement, such as exercise, without considering long‐term consequences. This aligns with the broader literature on impulsivity and decision‐making, where individuals with higher impulsive traits often exhibit a preference for immediate gratification,^45^, ^46^ even in contexts involving physical activity or exercise. Furthermore, the ICC analysis revealed agreement between the two delay discounting tasks across the entire sample, supporting the consistency of participants' decision‐making patterns across both monetary and exercise‐related contexts. However, while numerical differences in ICC values were observed between the groups, these differences were not statistically significant. This finding underscores the importance of cautious interpretation of numerical ICC differences and highlights the need for further studies to explore the factors that may influence ICC variability across different populations and contexts.

In contrast, the AN group showed a distinct pattern. The negative correlations observed indicate that individuals with AN, particularly those with less enjoyment of exercise, exhibited a greater willingness to delay immediate exercise rewards. This suggests that the reduced hedonic value of exercise in AN may contribute to a greater capacity to tolerate delays in gratification, as these individuals may be less emotionally engaged in exercise itself.^47^, ^48^ Rather than the enjoyment or intrinsic motivation typically driving exercise,^49^ the focus in AN may be more on the external goal of weight control or adhering to treatment protocols. This decreased exercise enjoyment, paired with the willingness to delay immediate rewards, may reflect a broader tendency toward self‐control, which is often seen in restrictive behaviors such as food intake regulation. These findings also resonate with the notion that the reduced emotional and hedonic response to exercise in AN may reinforce a greater focus on long‐term goals (such as weight control), rather than immediate rewards, further complicating the decision‐making process in this population.^50^

These findings underscore the importance of considering the psychological mechanisms at play in both reward processing and decision‐making in AN, where a combination of compulsivity, cognitive rigidity, and diminished reward sensitivity may shape delay discounting preferences. Moreover, the differential patterns observed between the general population and AN emphasize the need for more nuanced models of decision‐making that take into account the disorder‐specific features of eating behaviors, such as exercise and dietary restriction, which interact with broader psychological traits like impulsivity and compulsivity.

### Clinical implications

The findings of this study might have clinical implications for understanding the decision‐making processes in individuals with AN. The distinct patterns observed in delay discounting preferences between the two groups suggest that therapeutic interventions targeting impulsivity and compulsivity could be beneficial for individuals with AN. Specifically, the negative correlation between exercise enjoyment and delay discounting in the AN group may inform treatment strategies that focus on altering the hedonic value of exercise and its relationship to weight control. Clinicians might consider introducing interventions that aim to increase the intrinsic enjoyment of physical activity, thereby reducing the emphasis on delayed rewards tied to external goals, such as weight management. Moreover, these findings suggest that interventions specifically targeting compulsive exercise in individuals with AN could help reduce the overvaluation of delayed rewards tied to exercise and weight control. Given the rigidity and compulsivity often associated with exercise behaviors in AN, treatment plans might benefit from integrating techniques that enhance flexibility and diminish the immediate reinforcement of exercise routines.

Additionally, the positive correlations observed in the general population between impulsivity, compulsive exercise behaviors, and delay discounting highlight the need for interventions that address impulsivity and rigid decision‐making patterns, potentially improving self‐regulation and long‐term health outcomes. These results also suggest that tailored interventions that account for individual differences in reward sensitivity and delay discounting may be more effective in promoting sustainable changes in both exercise behaviors and broader treatment adherence. Overall, the integration of this information into clinical assessments could provide more personalized treatment plans, enhancing both short‐term and long‐term recovery outcomes.

### Limitations

Although this study has strengths, it is important to recognize its limitations when interpreting the results. The cross‐sectional design restricted our ability to assess only static relationships between constructs. Conducting longitudinal studies would provide a more comprehensive understanding of these elements over time. Furthermore, our inclusion of individuals from an inpatient setting may have influenced the severity of the results. Future studies should aim to collect data from a more diverse population to enhance generalizability and capture a wider range of demographic characteristics. Additionally, the observed discrepancies in ICC values between the overall AN group and its subgroups highlight the influence of sample size, data distribution, and statistical methodology on reliability estimates. Smaller subgroup samples may have amplified variability, while greater heterogeneity in the overall group may have masked subgroup‐specific differences, resulting in a higher ICC for the combined group. These factors underscore the need for cautious interpretation of subgroup‐level findings and emphasize the importance of ensuring robust sample sizes and methodological consistency in future research.

## Conclusion

In conclusion, our study sheds light on the complex interplay between the impulsive and compulsive characteristics of physical activity in individuals with AN compared to the general population. While confirming the existing literature on behavior associated with monetary rewards, our findings also underscore the different response patterns observed in individuals with AN, hinting at the presence of unique cognitive processes underlying their relationship with physical activity. By examining delay discounting in the context of monetary and physical activity rewards, this study provides valuable information on the motivational factors driving exercise participation in AN.

Future research could further explore the underlying mechanisms that link delay discounting, reward processing, and exercise behaviors, with implications for the development of targeted interventions aimed at promoting healthier attitudes toward exercise in individuals with AN. Moreover, future studies should include different diagnoses and conditions, looking for differences or similarities in the ED spectrum.

## Disclosure statement

On behalf of all authors, the corresponding author states that there is no conflict of interest.

## Author contributions

PM: conceptualization, methodology, data collection, formal analyses, data curation, original writing, and editing. LL: data collection, original writing and editing; PB: data collection and editing. VM: conceptualization, methodology, editing; EBucci: data collection and editing; AP: data collection and editing; CC: data collection and editing; AG: data collection and editing; EBonello: data collection and editing; FB: data collection and editing; PT: conceptualization, methodology and editing. All authors read and approved the final version.

## Supporting information


**Table S1.** Spearman correlation analyses between delay discounting tasks and psychological characteristics.

## Data Availability

Data supporting the findings of this study are available from the corresponding author upon reasonable request.
