# Supplementary material for: Distinct delay discounting patterns in anorexia nervosa: Comparing monetary and exercise rewards across clinical subgroups
Source: Psychiatry Clin Neurosci. 2025 Feb 10;79(5):266–73. doi: 10.1111/pcn.13802 (PMC12047058; doi:10.1111/pcn.13802)
Supplement: Supplementary file 1 — Table S1. Spearman correlation analyses between delay discounting tasks and psychological characteristics. [file PCN-79-266-s001.docx]

Supplementary Table 1. Spearman correlation analyses between delay discounting tasks and psychological characteristics.

|  | PA  Big | PA  Medium | PA  Small | PA  Average | M  Big | M  Medium | M  Small | M  Average |
| --- | --- | --- | --- | --- | --- | --- | --- | --- |
| CP | | | | | | | | |
| EDEQ  Global score | 0.93 | .105 | .155 | .109 | .123 | .165 | .170 | 196 |
| Avoidance | -.131 | -.009 | .011 | -.074 | .031 | .059 | -.017 | .023 |
| Weight control | -.031 | .017 | -.021 | -.009 | .071 | .054 | -.015 | .051 |
| Mood improvement | .257** | .300** | .279** | .239** | .124 | .117 | .027 | .096 |
| Lack of exercise enjoyment | -.074 | -.116 | -.072 | -.098 | -.005 | .033 | .071 | .015 |
| Exercise rigidity | .259* | .313** | .292** | .220** | .335** | .314** | .215* | .300* |
| CET  Global score | .124* | .204* | .188* | .151* | .192 | .214 | .162 | .190 |
| Positive urgency | .043 | .079 | .157 | .111 | .019 | .039 | .011 | .035 |
| Negative urgency | .090 | .136 | .107 | .127 | .136 | .121 | .015 | .109 |
| Lack of premeditation | .113 | .174 | .048 | .138 | .184 | .130 | .019 | .130 |
| Lack of perseverance | .091 | .182 | .171 | .149 | .100 | .078 | .052 | .082 |
| Sensation seeking | .049 | .082 | .047 | .055 | .075 | .110 | .074 | .098 |
| ANr | | | | | | | | |
| EDEQ  Global score | .352 | .276 | .144 | .245 | -.037 | -.202 | -.137 | -.098 |
| Avoidance | -.100 | -.039 | -.058 | -.044 | -.059 | -.129 | .106 | -.001 |
| Weight control | -.041 | .014 | -.149 | -.035 | .072 | -.216 | -.050 | -.046 |
| Mood improvement | .229 | .212 | .099 | .275 | -.095 | .239 | .228 | .140 |
| Lack of exercise enjoyment | -.400* | -.326* | -.222* | -.327* | .185 | -.003 | .011 | .131 |
| Exercise rigidity | .002 | .006 | .302 | .045 | -.042 | .160 | .272 | .173 |
| CET  Global score | -.107 | -.014 | -.099 | -.041 | -.007 | -.090 | .123 | .024 |
| Positive urgency | .151 | .023 | .273 | .115 | -.132 | .076 | .500** | .135 |
| Negative urgency | -.095 | -.156 | .074 | -.099 | -.083 | .045 | .559** | .135 |
| Lack of premeditation | -.034 | .058 | .239 | -.012 | .003 | -.050 | -.080 | -.014 |
| Lack of perseverance | .224 | .136 | .081 | .128 | .104 | -.088 | .051 | .115 |
| Sensation seeking | .096 | -.011 | .176 | .041 | -.136 | -.101 | .349* | -.016 |
| ANbp | | | | | | | | |
| EDEQ  Global score | -.132 | .006 | -.180 | -.191 | -.128 | .213 | .317 | .124 |
| Avoidance | -.190 | -.137 | -.120 | -.320 | -.037 | .158 | .085 | .067 |
| Weight control | -.345* | -.162 | .015 | -.324 | .096 | .311 | .165 | .211 |
| Mood improvement | -.194 | .090 | .239 | .014 | .031 | .119 | -.042 | .043 |
| Lack of exercise enjoyment | -.550** | -.594** | -.346** | -.479 | .050 | .093 | -.046 | .008 |
| Exercise rigidity | -.216 | -.122 | -.092 | -.316 | -.049 | .200 | .029 | .058 |
| CET  Global score | -.327 | -.173 | .048 | -.297 | .003 | .237 | .067 | .115 |
| Positive urgency | -.009 | .062 | .092 | .057 | -.243 | -.195 | -.039 | -.187 |
| Negative urgency | .026 | .116 | .239 | .265 | -.131 | -.312 | -.164 | -.254 |
| Lack of premeditation | -.226 | -.059 | .332 | -.009 | .278 | .273 | .007 | .201 |
| Lack of perseverance | -.226 | -.056 | .332 | -.009 | .276 | .273 | .281 | -.003 |
| Sensation seeking | -.039 | -.067 | .074 | -.031 | -.153 | -.093 | .102 | .986 |

The table reported Spearman’s rho. CP: community participant; AN: anorexia nervosa; r: restrictive; bp: binge-purge; EDEQ: eating disorder examination questionnaire; S-UPPS-P: short version of the impulsive behavior scale; CET: compulsive exercise test; * P < 0.05; ** p < 0.01
